# Association between different combination of measures for obesity and new-onset gallstone disease

**DOI:** 10.1371/journal.pone.0196457

**Published:** 2018-05-17

**Authors:** Tong Liu, Wanchao Wang, Yannan Ji, Yiming Wang, Xining Liu, Liying Cao, Siqing Liu

**Affiliations:** 1 Graduate school, North China University of Science and Technology, Tangshan, China; 2 Department of Hepatobiliary Surgery, Kailuan General Hospital Affiliated to North China University of Science and Technology, Tangshan, China; 3 School of Basic Medicine, Chende Medical University, Chende, China; Peking Union Medical College, CHINA

## Abstract

**Background:**

Body mass index(BMI) is a calculation index of general obesity. Waist circumference(WC) is a measure of body-fat distribution and always used to estimate abdominal obesity. An important trait of general obesity and abdominal obesity is their propensity to coexist. Using one single measure of obesity could not estimate persons at risk for GSD precisely.

**Objectives:**

This study aimed to compare the predictive values of various combination of measures for obesity(BMI, WC, waist to hip ratio) for new-onset GSD.

**Methods:**

We prospectively studied the predictive values of various combination of measures for obesity for new-onset GSD in a cohort of 88,947 participants who were free of prior gallstone disease, demographic characteristics and biochemical parameters were recorded.

**Results:**

4,329 participants were identified to have GSD among 88,947 participants during 713 345 person-years of follow-up. Higher BMI, WC and waist to hip ratio (WHtR) were significantly associated with higher risks of GSD in both genders even after adjustment for potential confounders. In males, the hazard ratio for the highest versus lowest BMI, WC, WHtR were 1.63(1.47~1.79), 1.53(1.40~1.68), 1.44(1.31~1.58), respectively. In females, the hazard ratio for the highest versus lowest BMI, WC, WHtR were 2.11(1.79~2.49), 1.85(1.55~2.22), 1.84(1.55~2.19), respectively. In male group, the combination of BMI+WC improved the predictive ability of the model more clearly than other combinations after adding them to the multivariate model in turn, while for females the best predictive combination was BMI+WHtR.

**Conclusions:**

Elevated BMI, WC and WHtR were independent risk factors for new-onset GSD in both sex groups after additional adjustment was made for potential confounders. In males, the combination of BMI+WC seemed to be the most predictable model to evaluate the effect of obesity on new-onset GSD, while the best combination in females was BMI+WHtR.

## Introduction

Gallstone disease (GSD) is a worldwide disease of digestive system with multifactorial origins[1]. Most of the reported prevalence of GSD in Western counties ranged from 10–15%[2]. Research shows it affects 10%-15% adult population, meaning 20–25 million adults in America are afflicted with GSD[3], with medical cost for the treatment of almost 6.2 billion annually[4]. In 1989, it was reported that the prevalence of gallstones in China was 6.29% among 100,000 people[5]. Although the mortality rate for GSD is low at 0.6%, an increased overall mortality, particularly from cancer and cardiovascular disease in GSD patients, resulting in a heavy burden on the economy and public health services [6].

Several studies have shown that advancing age, female gender and ethnicity are risk factors for gallstone disease which cannot be modified[7]. Also, previous studies have proven that obesity is closely related to GSD, although the evidence is not consistent[8–11]. Body mass index (BMI; in Kg/m^2^) is a calculation index of general obesity which is frequently analyzed in the studies that concerned the relation between obesity and GSD[1–3]. However, growing evidence of comparison studies suggested that abdominal obesity was a more important risk factor for stones formation than general obesity[12,13]. Waist circumference(WC; in cm) is a measure of body-fat distribution and always used to estimate abdominal obesity[14,15]. Furthermore, a former study has already demonstrated that WC is positively correlated with height, waist-to-height ratio (WHtR) may be a better measure for abdominal obesity which is adjusted for height[16].

An important trait of general obesity and abdominal obesity is their propensity to be coexist. This is owing partly to them sharing antecedent risk factors, also because one may directly predispose to the other[15]. Using one single measure of obesity could not estimate persons at risk for GSD precisely. It is highly doubted whether different combinations of measures for obesity have better predictive values than a single measure. Therefore, this study aimed to compare the predictive values of various combination of measures for obesity(BMI, WC, WHtR) for new-onset GSD.

## Methods

### Study design and population

Kailuan Study is a prospective population-based study in Kailuan community that is owned and managed by Kailuan Group in Tangshan city in northern China, and the study was designed to investigate risk factors for chronic disease. From July 2006 to October 2007, a total of 101,510 working and retired employees of the Kailuan Corporation underwent physical examinations including questionnaire survey, clinical examination, and laboratory testing at the Kailuan General Hospital and its 10 affiliated hospitals.

**Fig 1:**
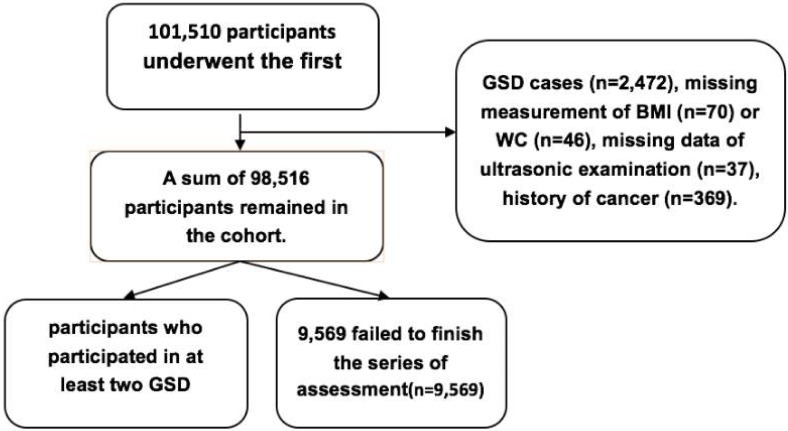
The procedure of participants screening.

In this study, we excluded participants who didn't meet the criterion: participants with a history of GSD (n = 2,472), participants without measurement of BMI (n = 70) or WC (n = 46), missing data of ultrasonic examination (n = 37), participants with a prior history of cancer (n = 369), leaving 98,516 participants remained in the study cohort. Subsequent biennial follow-up screenings for the 98,516 participants were conducted until 31 December, 2015 with repeated questionnaires and physical examinations. We analyzed all participants who participated in at least two GSD screening, 9,569 failed to finish the series of assessment. Therefore, the total amount of participants in this study was 88,947. Those who lost to follow-up during the 8-year period were older (51.09±11.07 years versus 50.82±12.09 years, *P*<0.001), and have higher levels of BMI(26.17±4.10 Kg/m^2^ versus 25.07±3.46 Kg/m^2^, *P* < 0.001) and exhibited higher total cholesterol concentrations(5.01±1.09 mmol/L versus 4.95±1.15 mmol/L, *P* < 0.001) and higher prevalence of hypertension(4497(0.47) versus 37682(42.4), *P* < 0.001), diabetes(871(9.1) versus 7632(8.6), *P*< 0.001). The Ethics Committee of Kailuan General Hospital approved our study, in compliance with the Declaration of Helsinki. All participants or their legal representatives signed informed consent forms. The details of participants screening were shown in the Fig 1.

### Type-B ultrasonic examination and assessment of gallstone disease

All participants were required to fast before examination, a panel of specialists examined the abdominal region (liver, gallbladder, pancreas and spleen in turn) of each participant, diagnosing GSD based on real-time ultrasound sonography (PHILIPS HD-15) with 3.5 MHz. GSD was diagnosed according to the following criteria: cholecystic cavity exhibited a hyperechoic mass, with a stable shape; the rear of the hyperechoic mass exhibited a clear acoustic shadow; after changing position, the hyperecholic mass moved in the direction of gravity. In patients undergoing cholecystectomy for cholelithiasis, Type-B ultrasound was used to describe the circumstances of the common bile duct and whether a gallstone existed inside the liver or not, and "gallbladder removed for cholelithiasis" was recorded in such circumstances[17].

### Physical examination and diagnostic criteria

Details of the collection of epidemiological data, anthropometric parameters were in accordance with the previous published articles by our group[18–20]. Basic data on height, weight, waist circumference, systolic blood pressure, diastolic blood pressure and other parameters were collected from all participants. BMI was measured as body weight in kilograms divided by the square of the body height in meters and was grouped into three categories, normal(BMI<24.0Kg/m^2^), overweight(24.0<BMI≤28.0 Kg/m^2^), general obesity(BMI≥28.0 Kg/m^2^). WC was measured with a tape measure midway between the lowest rib and the pelvis in position of expiration and was categorized into three groups by its tertiles. WHtR was measured as WC in cm divided by measured height in cm and was categorized into three groups by its tertiles. The diagnostic criteria for hypertension was in accordance with the World Health Organization: systolic blood pressure ≥140mmHg and/or diastolic blood pressure≥90mmHg, diagnosed hypertension previously, had taken or was taking antihypertensive drugs. Smoking was defined as having smoked at least one cigarette per day on average for at least one year. Drinking status was defined as having 100ml/day(alcohol contents>50%) of alcohol for more than one year. Exercise was defined as having≥3 times weekly with each time lasting at least 30 minutes.

### Laboratory examination parameters

Blood samples, after an overnight fast, were collected from cubital vein for 5ml at 7–9 am in the physical examination day. After separating and extracting serum, a same group of laboratory technicians tested the biochemistry analyzer (Hitachi 7600, Tokyo, Japan). The indicator included fasting blood glucose (FBG, in mmol/L), total cholesterol(TC, in mmol/L), triglyceride (TG, in mmol/L). The diagnostic criteria for diabetes was in accordance with the World Health Organization: FBG≥7.0mmol/L, previously diagnosed diabetes, had undergone or was undergoing hypoglycaemic therapy.

### Statistical analysis

Data were input by data entry clerk of individual hospitals and transferred to Oracle 10g database located in Kailuan Hospital through Internet. Data in normal distribution were presented as mean ± standard deviation, and compared using one-way analysis of variance(ANOVA). The skewed distribution of data was described by median (interquartile range) and analyzed by the nonparametric. Categorical variables were described by percentage (N%) and chi-squared test was applied for the comparison of categorical variables. Pearson-years of follow-up were calculated as the time from baseline assessment to either the diagnosis of GSD or censoring, or end of follow-ups (December 31, 2015), whichever happened first. The incidence rates were calculated by dividing the number of events by person-years of follow-up. Cox proportional hazard model was used to calculate hazard ratios (HRs) and their 95% confidence intervals (CIs) for GSD. We created three multivariate-adjusted models. Model 1 was an univariate model (GSD status were defined as dependent variables, three prespecified categories of BMI, WC, WHtR were served as independent variables, respectively.). Model 2 was adjusted for age based on model 1, Model 3 was further adjusted for age, TC, TG, diabetes(yes/no), hypertension(yes/no), current smoker(yes/no), drinking status (yes/no), physical activity (yes/no). The -2log L (likelihood ratio) and AIC (Akaike Information Criterion) were used as measures of the improvement of goodness of fit among models that added different combinations of measures for obesity to the multivariate model (Multivariate model included age, TG, TG, BMI, current smoker, drinking status, diabetes, hypertension, physical activity). Males and females were analyzed separately. Reported *p* values are two-sides, and all statistical computations were done using the SAS 9.4 statistical software.

## Results

### Analysis of general characteristics of participants stratified by sex

4,329 participants were identified to have GSD among 88,947 participants during 713,345 person-years of follow-up (The mean follow-up is 8.11±1.17 years). The overall incidence per 1000 person-years of GSD was 6.02 (4,329/713,345). The general characteristics of the participants stratified by sex were shown in Table 1. Males were older in age, larger in WC and higher in BMI, systolic blood pressure (SBP, in mmHg), diastolic blood pressure (DBP, in mmHg), FBG, and were more likely to have hypertension, diabetes. Male participants were associated with higher prevalence of current smoking, drinking status, and higher percentage of physical activity>4 times/week. While females were associated with higher concentrations of TC and TG.

**Table 1:** Baseline characteristics of the population stratified by sex.

	Male	Female	Total	F/*X*^*2*^	P value
No. of participants	70 172	18 775	88 947	—	—
Age (Year)	51.44±12.21	48.51±11.32	50.82±12.09	F = 877.29	<0.001
SBP (mmHg)	131.90±20.30	124.35±20.89	130.31±20.66	F = 2014.10	<0.001
DBP (mmHg)	84.42±11.63	79.37±11.00	83.36±11.68	F = 2845.46	<0.001
WC(cm)	87.99±9.51	82.91±10.67	86.92±9.98	F = 4012.24	<0.001
BMI(Kg/m^2^)	25.19±3.37	24.65±3.78	25.07±3.46	F = 354.69	<0.001
FBG(mmol/L)	5.49±1.63	5.30±1.60	5.45±1.62	F = 209.95	<0.001
TC(mmol/L)	4.95±1.16	4.98±1.08	4.95±1.15	F = 10.38	<0.001
TG(mmol/L)	1.18(0.81~1.74)	1.30(0.92~2.00)	1.27(0.89~1.94)	*X*^*2*^ = 642.39	<0.001
Current smoker(%)	26993(38.47)	248(1.32)	27241(30.6)	*X*^*2*^ = 9619.42	<0.001
Drinking status(%)	15779(22.49)	87(0.46)	15866(17.8)	*X*^*2*^ = 4901.75	<0.001
Diabetes(%)	6223(8.87)	1409(7.50)	7632(8.6)	*X*^*2*^ = 35.10	<0.001
Hypertension(%)	31816(35.77)	5866(31.24)	37682(42.4)	*X*^*2*^ = 1205.40	<0.001
Physical activity(%)	11063(15.77)	2450(13.05)	13513(15.2)	*X*^*2*^ = 84.82	<0.001

SBP: systolic blood pressure, DBP: diastolic blood pressure, WC: waist circumference, BMI: body-mass index, FBG: fasting blood glucose, TC: total cholesterol, TG: triglycerides.

### Cox proportional hazard model of BMI, WC and WHtR for gallstone disease

After adjustment was made for traditional risk factors including age(year), TC(mmol/L), TG(mmol/L), diabetes(yes/no), hypertension(yes/no), current smoker(yes/no), drinking status(yes/no), physical activity(yes/no), the hazard ratios(HRs) of GSD indicated a clear trend for each sex group, where values increased as BMI, WC, WHtR increased. In males, the hazard ratios increased from 1.34(1.23~1.45) at 24.0<BMI≤28.0 Kg/m^2^ to 1.63(1.47~1.79) in BMI≥28 Kg/m^2^ (BMI<24Kg/m^2^ served as the reference group), 1.27(1.15~1.40) at 84.0<WC≤91.0 cm to 1.53(1.40~1.68) in WC≥91.0 cm (WC<84.0 cm served as the reference group) and 1.20(1.09~1.32) at 0.49<WHtR≤0.54 to 1.44(1.31~1.58) in WHtR≥0.54 (WHtR<0.49 served as the reference group). The trend of hazard ratios in females was similar with it in males. And the detailed results were shown in Table 2, Table 3 and Table 4.

**Table 2:** Hazard ratios (HRs) and 95% confidence interval(CI) for risk of new-onset gallstone disease according to BMI subgroups.

	Men			Women		
	BMI<24	24≤BMI<28	BMI≥28	BMI<24	24≤BMI<28	BMI≥28
Cases of GSD	963	1523	786	329	423	305
Person-years	210389	246415	106017	70939	54000	25584
Model 1	Ref	1.35(1.25–1.47)	1.63(1.48–1.79)	Ref	1.68(1.46–1.94)	2.57(2.20–3.00)
Model 2	Ref	1.36(1.26–1.48)	1.68(1.53–1.85)	Ref	1.49(1.29–1.73)	2.23(1.90–2.62)
Model 3	Ref	1.34(1.23–1.45)	1.63(1.47–1.79)	Ref	1.46(1.26–1.69)	2.11(1.79–2.49)

Note: Model 1, an univariate analysis.

Model 2, adjusted for age(year).

Model 3, adjusted for age(year), total cholesterol (mmol/L), triglycerides (mmol/L), current smoker(yes/no), drinking status(yes/no), diabetes(yes/no), hypertension(yes/no), physical activity (yes/no).

**Table 3:** Hazard ratios (HRs) and 95% confidence interval(CI) for risk of new-onset gallstone disease according to WC tertiles.

	Men			Women		
	WC<84	84≤WC<91	WC≥91	WC<78	78≤WC<87	WC≥87
Cases of GSD	751	1037	1484	187	364	506
Person-years	177847	183035	201938	47296	52897	50330
Model 1	Ref	1.34(1.22–1.47)	1.74(1.59–1.90)	Ref	1.72(1.45–2.06)	2.50(2.11–2.95)
Model 2	Ref	1.29(1.18–1.42)	1.57(1.44–1.72)	Ref	1.48(1.23–1.77)	1.95(1.64–2.33)
Model 3	Ref	1.27(1.15–1.40)	1.53(1.40–1.68)	Ref	1.44(1.20–1.73)	1.85(1.55–2.22)

Note: Participants were divided by waist circumstance (WC) tertiles in each sex.

Model 1, an univariate analysis.

Model 2, adjusted for age(year).

Model 3, adjusted for age(year), total cholesterol (mmol/L), triglycerides (mmol/L), current smoker(yes/no), drinking status(yes/no), diabetes(yes/no), hypertension(yes/no), physical activity (yes/no).

**Table 4:** Hazard ratios (HRs) and 95% confidence interval(CI) for risk of new onset gallstones disease according to WHtR tertiles.

	Men			Women		
	WHtR<0.49	0.49≤WHtR<0.54	WHtR≥0.54	WHtR<0.49	0.49≤WHtR<0.54	WHtR≥0.54
Cases of GSD	724	1146	1402	213	305	539
Person-years	169036	204826	188958	52771	45443	52309
Model 1	Ref	1.30(1.19–1.43)	1.73(1.58–1.89)	Ref	1.64(1.38–1.96)	2.50(2.13–2.93)
Model 2	Ref	1.22(1.11–1.34)	1.48(1.35–1.62)	Ref	1.42(1.19–1.70)	1.94(1.63–2.29)
Model 3	Ref	1.20(1.09–1.32)	1.44(1.31–1.58)	Ref	1.39(1.16–1.66)	1.84(1.55–2.19)

Note: Participants were divided by waist-to-height ratio(WHtR) tertiles in each sex (Both sexes have the same cut-off value).

Model 1, an univariate analysis.

Model 2, adjusted for age(year).

Model 3, adjusted for age(year), total cholesterol (mmol/L), triglycerides (mmol/L), current smoker(yes/no), drinking status(yes/no), diabetes(yes/no), hypertension(yes/no), physical activity (yes/no).

### The improvements of fitness for different combinations of measures for obesity

The likelihood ratio test showed that all combinations of measures for obesity could improved the models' goodness of fit when adding them to the traditional multivariate model which included age, TC, TG, current smoker, drinking status, diabetes, hypertension and physical activity. In male group, the combination of BMI+WC improved the predictive ability of the model more clearly than other combinations after adding them to the multivariate model in turn, while for females the best predictive combination was BMI+WHtR. The detailed results were shown in Table 5.

**Table 5:** Increases in goodness of fit by progressively adding to multivariate model.

	Men			Women	
	-2 Log L	AIC		-2 Log L	AIC
Multivariate (Ref)	70918	70936	Multivariate (Ref)	20102	20120
Multivariate +BMI	70818	70840	Multivariate +BMI	20026	20048
Multivariate +WC	70830	70852	Multivariate +WC	20057	20079
Multivariate +WHtR	70857	70879	Multivariate +WHtR	20054	20076
Multivariate +BMI+WC^#^	70795	70831	Multivariate +BMI+WC	20019	20045
Multivariate +BMI+WHtR	70810	70836	Multivariate +BMI+WHtR*	20018	20044
Multivariate +WC+WHtR	70830	70856	Multivariate +WC+WHtR	20052	20078

Note: -2log L: likelihood ratio, AIC:Akaike Information Criterion.

The difference of—2 Log L value of 3.8 corresponds with *P* value of 0.05, 6.6 to 0.01, and 10.8 to 0.001.

Multivariate model included age(year), total cholesterol (mmol/L), triglycerides (mmol/L), current smoker(yes/no), drinking status(yes/no), diabetes(yes/no), hypertension(yes/no), physical activity (yes/no).

BMI: body mass index, WC: waist circumstance, WHtR: waist-to-height ratio.

#: The best predictable model in men.

*: The best predictable model in women.

## Discussion

Epidemiological studies have reported a steadily increase in the incidence of chronic diseases such as hypertension, diabetes, coronary heart disease and gallstones disease with increasing BMI, WC or WHtR[16,21]. BMI, a useful measure of overall obesity, dose not distinguish between fat and lean body mass, may not served as a perfect measure of obesity, particularly in older adults[22]. Therefore, abdominal obesity must be taken into consideration when studies specialized in relation between adiposity and GSD. Recently, researchers have founded that magnetic resonance imaging and computed tomography showed a simple measurement such as WC was the best anthropometric correlate of the amount of visceral adipose tissue[23]. Also, growing evidence of comparison studies suggested that WHtR was the best predictor for metabolic diseases[24]. It is doubted whether predictive values varied among different combinations of measures for obesity (BMI, WC, WHtR) in the same model which evaluate the effect of obesity on new-onset GSD.

In this large prospective cohort study, we found that higher concentrations of BMI, WC and WHtR were significantly associated with higher risks of GSD in both sex groups even after adjustment was made for potential confounders. Study conducted in the US had found women with BMI≥32.0Kg/m^2^ could expect a sixfold increase in the risk of GSD compared to those at BMI<20.0Kg/m^2^, while studies concerned about Japanese men or Taiwanese men failed to find such a relationship[1,20,21]. However, an inverse relationship between BMI and GSD had been reported in several epidemiologic studies[25–27]. Abdominal obesity was also shown to be a powerful predictor of GSD. Heaton KW et al reported that the waist to hip ratio, not body mass index, was significantly associated with GSD[28]. A population-based study of 29,847 US men in which ultrasonography was used to assess GSD confirmed a positive relation between WC and new-onset GSD[16]. But, other studies failed to find such an association[29,30]. Those discrepancies may be due to different epidemiological approaches, ethnicity and genders.

There has been a huge interest in the development of effective strategies to recognize persons at risk for GSD. Traditional risk factors such as age, sex, obesity, diabetes mellitus have been used in several studies that concerned about the risks of GSD worldwide[1–3]. The relationship between obesity and GSD is a major public health concern. New methods that evaluate the obesity should be tested in prospective cohort studies for effects in prediction models. Improving our ability to assess risk more accurately so that the most appropriate follow-up and care can be provided. As with our results, the combination of BMI+WC seemed to be the most predictable model for new-onset GSD in male participants, while the best combination in females were BMI+WHtR. It was not surprising to find that BMI was included in both sex groups, indicating that general obesity was essential for stones formation in both sex groups. But the measures of abdominal obesity were not the same. WC was found to be a better measure in males. As for female, WHtR was the best measure. Different obesity types as well as abdominal fat distribution between men and women may explain this discrepancy[31].

The possible pathogenesis for the close association between obesity and GSD are complex and not fully understood. However, cholesterol-supersaturated bile obese participants and the gallbladder volume was observed to be larger in obese patients. Also, the activities of the rate-limiting enzyme of cholesterol synthesis that could cause cholesterol supersaturation and secreted into the bile duct, thus contributing to gallstone formation.

The composition of gallstones in Asian is considered to be mainly pigment stones, which is different from that of Western countries[32–34]. Nevertheless, the composition might change with dietary diet and lifestyle. Cholesterol stones that considered to be related to obesity, diabetes, or dyslipidemia, sex hormone estrogen are common among Western population. In contrast, pigment stones that may be related to bacterial infection, hemolysis, or liver disease rather than hormone factors are common in Asia[35]. The changes in diet and lifestyle have been linked to the increased prevalence of gallstones as exemplified by Japan: The frequency of GSD doubled in the late 1940s, in association with the occurrence a change in stone's composition from pigment to cholesterol and a reversal in the sex ratio, which women become the predominant gender diagnosed, was also seen[36]. In our study, sex and obesity are observed to be related to GSD. Could this mean the composition of stones have also changed in China?

The strength of this study was: the gallbladder was unselectively evaluated by ultrasonography in a defined population, its large sample size and high participation rates during an eight-year period. However, several limitations should be noticed in our study. First, ultrasonography is regarded as a safe, accurate, and convenient tool to evaluate the presence of gallstone disease in epidemiological surveys, but stone types cannot be provided. Second, female gender is an established risk factor for GSD, and pregnancies and the frequency of oral contraceptives were founded to be positively related to GSD. however, there are no data considering the number of pregnancies and the frequency of oral contraceptives. Third, the majority of the participants were from the Kailuan Mine Corporation, with more males than females in the study, but the bias concerning sex can be minimized as men and women were studied separately. Finally, the characteristics of the general participants significantly differed from those of non-respondents, indicating that the participants who did not complete the follow up might have severe GSD.

## Conclusions

Our study corroborated that elevated BMI, WC and WHtR were independent risk factors for new-onset GSD in both sex groups after additional adjustment was made for potential confounders. In males, the combination of BMI+WC seemed to be the most predictable model to evaluate the effect of obesity on new-onset GSD, while the best combination in females were BMI+WHt.

## References

[1] Chen CH, Huang MH, Yang JC, Nien CK, Etheredge GD, Yang CC, et al Prevalence and risk factors of gallstone disease in an adult population of Taiwan: an epidemiological survey. J Gastroenterol Hepatol. 2006 11;21(11):1737–43. doi: 10.1111/j.1440-1746.2006.04381.x 16984599 10.1111/j.1440-1746.2006.04381.x

[2] Zhu L, Aili A, Zhang C, Saiding A, Abudureyimu K. Prevalence of and risk factors for gallstones in Uighur and Han Chinese. World J Gastroenterol. 2014 10 28;20(40):14942–9. doi: 10.3748/wjg.v20.i40.14942 25356055 10.3748/wjg.v20.i40.14942 PMC4209558

[3] Everhart JE, Khare M, Hill M, Maurer KR. Prevalence and ethnic differences in gallbladder disease in the United States. Gastroenterology. 1999 9;117(3):632–9. 10464139 10.1016/s0016-5085(99)70456-7

[4] Everhart JE, Ruhl CE. Burden of digestive diseases in the United States part I: overall and upper gastrointestinal diseases. Gastroenterology. 2009 2;136(2):376–86. doi: 10.1053/j.gastro.2008.12.015 19124023 10.1053/j.gastro.2008.12.015

[5] Zhuang X, Li U. Epidemiologic study of risk factors of gallstone. Zhonghua Liu Xing Bing Xue Zazhi 1999; 20: 181–183 10682533

[6] Ruhl CE, Everhart JE. Gallstone disease is associated with increased mortality in the United States. Gastroenterology. 2011 2;140(2):508–16. doi: 10.1053/j.gastro.2010.10.060 21075109 10.1053/j.gastro.2010.10.060 PMC3060665

[7] Shaffer EA. Gallstone disease: Epidemiology of gallbladder stone disease. Best Pract Res Clin Gastroenterol. 2006;20(6):981–96. Review. doi: 10.1016/j.bpg.2006.05.004 17127183 10.1016/j.bpg.2006.05.004

[8] Erlinger S. Gallstones in obesity and weight loss. Eur J Gastroenterol Hepatol. 2000 12;12(12):1347–52. Review. 11192327 10.1097/00042737-200012120-00015

[9] Amaral JF, Thompson WR. Gallbladder disease in the morbidly obese. Am J Surg. 1985 4;149(4):551–7. 3985293 10.1016/s0002-9610(85)80055-6

[10] Johansen C, Chow WH, Jørgensen T, Mellemkjaer L, Engholm G, Olsen JH. Risk of colorectal cancer and other cancers in patients with gall stones. Gut. 1996 9;39(3):439–43. 8949651 10.1136/gut.39.3.439 PMC1383353

[11] Tsai CJ, Leitzmann MF, Willett WC, Giovannucci EL. Prospective study of abdominal adiposity and gallstone disease in US men. Am J Clin Nutr. 2004 7;80(1):38–44. doi: 10.1093/ajcn/80.1.38 15213025 10.1093/ajcn/80.1.38

[12] Attili AF, Capocaccia R, Carulli N, Festi D, Roda E, Barbara L, et al Factors associated with gallstone disease in the MICOL experience. Multicenter Italian Study on Epidemiology of Cholelithiasis. Hepatology. 1997 10;26(4):809–18. doi: 10.1002/hep.510260401 9328297 10.1002/hep.510260401

[13] Dubrac S, Parquet M, Blouquit Y, Gripois D, Blouquit MF, Souidi M, et al Insulin injections enhance cholesterol gallstone incidence by changing the biliary cholesterol saturation index and apo A-I concentration in hamsters fed a lithogenic diet. J Hepatol. 2001 11;35(5):550–7. 11690699 10.1016/s0168-8278(01)00180-5

[14] Matta J, Carette C, Rives Lange C, Czernichow S. [French and worldwide epidemiology of obesity]. Presse Med. 2018 4 24 pii: S0755-4982(18)30186-6. doi: 10.1016/j.lpm.2018.03.023 29703570 10.1016/j.lpm.2018.03.023

[15] Guglielmo D, Hootman JM, Murphy LB, Boring MA, Theis KA, Belay B, et al Health Care Provider Counseling for Weight Loss Among Adults with Arthritis and Overweight or Obesity—United States, 2002–2014. MMWR Morb Mortal Wkly Rep. 2018 5 4;67(17):485–490. doi: 10.15585/mmwr.mm6717a2 29723172 10.15585/mmwr.mm6717a2 PMC5933870

[16] Tsai CJ, Leitzmann MF, Willett WC, Giovannucci EL. Prospective study of abdominal adiposity and gallstone disease in US men. Am J Clin Nutr. 2004 7;80(1):38–44. doi: 10.1093/ajcn/80.1.38 . 15213025 10.1093/ajcn/80.1.38

[17] Kono S, Shinchi K, Todoroki I, Honjo S, Sakurai Y, Wakabayashi K, et al Gallstone disease among Japanese men in relation to obesity, glucose intolerance, exercise, alcohol use, and smoking.Scand J Gastroenterol. 1995 4;30(4):372–6. Erratum in: Scand J Gastroenterol.1995 Dec;30(12):1228. 7610355 10.3109/00365529509093293

[18] Wu S, Huang Z, Yang X, Zhou Y, Wang A, Chen L, et al Prevalence of ideal cardiovascular health and its relationship with the 4-year cardiovascular events in a northern Chinese industrial city. Circ Cardiovasc Qual Outcomes. 2012 7 1;5(4):487–93. doi: 10.1161/CIRCOUTCOMES.111.963694 22787064 10.1161/CIRCOUTCOMES.111.963694

[19] Jia Z, Zhou Y, Liu X, Wang Y, Zhao X, Wang Y, et al Comparison of different anthropometric measures as predictors of diabetes incidence in a Chinese population. Diabetes Res Clin Pract. 2011 5;92(2):265–71. doi: 10.1016/j.diabres.2011.01.021 21334088 10.1016/j.diabres.2011.01.021

[20] Zhang Q, Zhou Y, Gao X, Wang C, Zhang S, Wang A, et al Ideal cardiovascular health metrics and the risks of ischemic and intracerebral hemorrhagic stroke. Stroke. 2013 9;44(9):2451–6. doi: 10.1161/STROKEAHA.113.678839 23868276 10.1161/STROKEAHA.113.678839

[21] Boggs DA, Rosenberg L, Cozier YC, Wise LA, Coogan PF, Ruiz-Narvaez EA, et al General and abdominal obesity and risk of death among black women. N Engl J Med. 2011 9 8;365(10):901–8. doi: 10.1056/NEJMoa1104119 21899451 10.1056/NEJMoa1104119 PMC3206314

[22] Sebo P, Beer-Borst S, Haller DM, Bovier PA. Reliability of doctors' anthropometric measurements to detect obesity. Prev Med. 2008 10;47(4):389–93. doi: 10.1016/j.ypmed.2008.06.012 18619998 10.1016/j.ypmed.2008.06.012

[23] Després JP, Lemieux I, Prud'homme D. Treatment of obesity: need to focus on high risk abdominally obese patients. BMJ. 2001 3 24;322(7288):716–20. 11264213 10.1136/bmj.322.7288.716 PMC1119905

[24] Schneider HJ, Friedrich N, Klotsche J, Pieper L, Nauck M, John U, et al The predictive value of different measures of obesity for incident cardiovascular events and mortality. J Clin Endocrinol Metab. 2010 4;95(4):1777–85. doi: 10.1210/jc.2009-1584 20130075 10.1210/jc.2009-1584

[25] Festi D, Dormi A, Capodicasa S, Staniscia T, Attili AF, Loria P, et al Incidence of gallstone disease in Italy:results from a multicenter, population-based Italian study (the MICOL project).World J Gastroenterol. 2008 9 14;14(34):5282–9. doi: 10.3748/wjg.14.5282 18785280 10.3748/wjg.14.5282 PMC2744058

[26] Salinas G, Velásquez C, Saavedra L, Ramírez E, Angulo H, Tamayo JC, et al Prevalence and risk factors for gallstone disease. Surg Laparosc Endosc Percutan Tech. 2004 10;14(5):250–3. 15492651 10.1097/00129689-200410000-00003

[27] Lu SN, Chang WY, Wang LY, Hsieh MY, Chuang WL, Chen SC, et al Risk factors for gallstones among Chinese in Taiwan. A community sonographic survey. J Clin Gastroenterol. 1990 10;12(5):542–6. 2229997 10.1097/00004836-199010000-00011

[28] Heaton KW, Fem Braddon, Emmett PM, Mountford RA, Hughes AP, Bolton CH, Ghost S.Why do men get gallstones-roles of abdominal fat and hyperrinsulinemia [J].European Journal Of gastroenterology and hepatology.1991,Vol.3(No.10): 745–751

[29] Haffner SM, Diehl AK, Stern MP, Hazuda HP. Central adiposity and gallbladder disease in Mexican Americans. Am J Epidemiol. 1989 3;129(3):587–95. 2916552 10.1093/oxfordjournals.aje.a115171

[30] Gonzalez Villalpando C, Rivera Martinez D, Arredondo Perez B, Martinez Diaz S, Gonzalez Villalpando ME, Haffner SM, Stern MP. High prevalence of cholelithiasis in a low income Mexican population: an ultrasonographic survey. Arch Med Res. 1997 Winter;28(4):543–7. 9428581

[31] Fischer J, Johnson MA. Low body weight and weight loss in the aged. J Am Diet Assoc. 1990 12;90(12):1697–706. Review. 2131340

[32] Tsai CJ, Leitzmann MF, Willett WC, Giovannucci EL. Prospective study of abdominal adiposity and gallstone disease in US men. Am J Clin Nutr. 2004 7;80(1):38–44. doi: 10.1093/ajcn/80.1.38 15213025 10.1093/ajcn/80.1.38

[33] Angelico F, Del Ben M, Barbato A, Conti R, Urbinati G. Ten-year incidence and natural history of gallstone disease in a rural population of women in central Italy. The Rome Group for the Epidemiology and Prevention of Cholelithiasis (GREPCO). Ital J Gastroenterol Hepatol. 1997 6;29(3):249–54. 9646217

[34] Chen JY, Hsu CT, Liu JH, Tung TH. Clinical predictors of incident gallstone disease in a Chinese population in Taipei, Taiwan. BMC Gastroenterol. 2014 4 28;14:83 doi: 10.1186/1471-230X-14-83 24775330 10.1186/1471-230X-14-83 PMC4006445

[35] Chen CY, Lu CL, Huang YS, Tam TN, Chao Y, Chang FY, Lee SD. Age is one of the risk factors in developing gallstone disease in Taiwan. Age Ageing. 1998 7;27(4):437–41. 9883999 10.1093/ageing/27.4.437

[36] Kameda H, Ishihara F, Shibata K, Tsukie E. Clinical and nutritional study on gallstone disease in Japan. Jpn J Med. 1984 5;23(2):109–13. 6328072 10.2169/internalmedicine1962.23.109

